# Maritime welfare facilities - utilization and relevance for the compensation of shipboard stress

**DOI:** 10.1186/s12995-019-0231-3

**Published:** 2019-04-11

**Authors:** Marcus Oldenburg, Hans-Joachim Jensen

**Affiliations:** Institute for Occupational and Maritime Medicine Hamburg (ZfAM), University Medical Centre Hamburg-Eppendorf (UKE), Seewartenstrasse 10, 20459 Hamburg, Germany

**Keywords:** Maritime, Welfare facilities, Compensation, Seafarer

## Abstract

**Background:**

Maritime welfare institutions enable crew members of ships to use different recreation facilities ashore during their work assignments aboard. This study analyses the shipcrew members’ need and their usage behavior of maritime welfare facilities that can be visited free of charge while their ship is in port.

**Methods:**

A medically trained scientist interviewed 316 seafarers during 22 ship voyages. In addition, interviews were performed in 8 stations of the German Seamen’s Missions.

**Results:**

Port stay of the vessels accounted for 43.6% of the entire voyage duration. 279 seafarers (88.3%) stated having had opportunities for shore leave in order to visit maritime welfare facilities. Officers less often stated possibilities for shore leave than ratings (OR 0.40; 95%CI 0.24–0.67). The main reasons for shore leave were to contact family and friends and distraction from the everyday work on board. Short stays in port, a high workload and limited opportunities for reaching the welfare facilities were main obstacles to shore leave. Furthermore, several seafarers complained about poor information on board about the facilities. Among the various welfare institutions, a “very high importance” was attributed to Seamen’s Missions and religious facilities (40% of the non-European seafarers - especially from Asia - and approx. 10% of the Europeans).

**Conclusion:**

This study reveals sufficient time periods for seafarers to leave the vessel in port, mainly depending on the number of terminals to be called at. During the vessels’ port stay, a reduced workload for the shipping crew can be achieved by transferring several routine tasks to shoreside personnel. Furthermore, more attention should be paid to the accessibility of the welfare facilities and to better information about their offers. Measures should be taken to facilitate visits to maritime welfare facilities as an important refuge for seafarers.

## Background

Long contracts, changing work shifts, permanent influence of physical parameters over 24 h per day are only a few examples representing the high psychophysical stress to which seafarers are permanently exposed on board [1–3]. These stressors seem to be especially prevalent on feeder vessels which are suppliers and distributors in the North Sea and Baltic Sea for large container vessels and seaports. In some ports, several terminals have to be served for loading and unloading operations. This often leads to a very high workload for ship’s crews because they have to manage numerous professional tasks (e.g. arrival and departure manoeuvres, loading and unloading) within a short time range [4, 5].

The high workload often elicits psychophysical fatigue, especially for crews on feeder ships [6, 7]; this is even more so when the individual seafarer’s psychophysical resources are exhausted. Their personal resources can be strengthened by distracting their thoughts from their work routines and through leisure time activities outside of the vessel [8]. According to Barnett and Hyde [9], the possibility of changing from a working role to a leisure time role enhances the individual’s competence in dealing with occupational stress and can mobilise further personal resources. This is also applicable for seafarers when visiting Seamen’s Facilities during port stay. It helps them to distance themselves from the strict hierarchical roles on board for some hours and allows them to experience relief and relaxation through social contacts and care [9].

Social welfare institutions like Seamen’s Missions, religious facilities, sport clubs or bars are often situated close to the ports. In this context, spiritual care - especially in welfare facilities - is of great importance [10]. To date, there are hardly any scientific surveys available that deal with communication within a multinational shipboard crew and with the importance of maritime welfare facilities. Only two studies discussed the social integration of East Asian seafarers in the “host society” [11] or the cultural implications [12]. There are no findings on the frequency and motivation of seafarers to visit welfare facilities. To improve the importance of maritime welfare facilities, however, it is necessary to understand the needs of the shipboard crews and their expectations towards these organisations.

Normally, seafarers work on board for several months. As their life and work is characterized by the environment on the ship over a long period, port stays have a special importance for recreation [13]. During port time, it is theoretically possible to leave the vessel and spend a part of the individual leisure time outside of the vessel. This requires, however, compatibility with the professional obligations.

So far, there are no studies about the average duration of vessels’ port stays in the North Sea and Baltic Sea and about the seafarers’ options to visit welfare facilities in the ports. This study aims to demonstrate which maritime welfare facilities are attended by seafarers during their port stay and to what extent. A particular interest of the study is also to explore which factors hinder the seafarers from visiting these facilities. Possible ethnic differences in the importance and use of leisure facilities will also be addressed.

## Methods

A medically trained scientist accompanied 22 sea voyages (aboard 16 feeder ships in coastal trade and 6 large container vessels in global trade) and recorded port stays as well as terminals called at per port. Beginning and end of the port stays were defined with docking and undocking of the ship and documented to the precise minute.

A total of 316 of 365 seafarers aboard the 22 included vessels participated in this study (participation rate 86.6%). The examiner interviewed them by means of a questionnaire concerning their attitude towards and usage behaviour regarding maritime welfare facilities in port. Participation in this study was entirely voluntary and the data were collected pseudonymously. All participants gave their informed consent before taking part in this study. The study was approved by the Ethics Committee of the Hamburg Medical Association (no PV4395).

At the time of our interview, the seafarers’ previous stay on board had lasted on average 106 days (from 5 to 380 days). The study sample was composed of 147 Europeans (46.5%) and 169 Southeast Asians (53.5%, mainly from the Philippines). The average age of the exclusively male seafarers was 38.5 years (±11.8 years), taking into account that the officers were a little older. 213 (66.6%) of the participants had children.

Concerning the professional stratification, a differentiation was made between the 121 officers (38.3%; 66 nautical and 55 technical officers) and the 195 ratings (61.7%). While 191 of the examined seafarers (60.4%) were working on 16 feeder ships in coastal trade (North range: North Sea incl. English Channel, Baltic Sea), 125 seafarers (39.6%) were signed on to 6 large container vessels in global trade. Sociodemographic and occupational data did not differ between the study subgroups working in the North range and on worldwide shipping routes.

### Statistical analysis

Data analysis was performed with SPSS for Windows (version 20.0, SPSS GmbH Software, Munich, Germany). The Pearson Chi-square test was applied to compare frequencies between groups. The crude odds ratio (OR) including 95% confidence intervals was calculated with binary logistic regression. Age and duration of stay on board at the time of our interview were added for adjustment reasons. All indicated *p*-values were two-sided, and a p-value of < 0.05 was regarded as statistically significant.

## Results

### Port stays

During the 22 ship’s voyages accompanied by the examiner, 90 ports were served in nearly 180 days. This corresponds to 43.6% of the total travel time. The number of the terminals served varied significantly between the different voyages. For example, a particularly high number [6] had to be called at in the port of Hamburg during the first voyage (Table 1).

**Table 1:** Voyage and proportion of time spent on port stays during the investigated 22 sea voyages

Ship‘s journey	Ports		Cumulative termi-nals	Cumulative duration (days: hours) (% during examination voyage)	
	*Sailing list*	*n*	*per 7 days*	*Port*	*Total*
1	Hamburg (GER), Kottka (FI), Rauma (SE), Bremerhaven (GER), Hamburg (GER)	5	11	3: 14 (35.0%)	10: 6 (100%)
2^a^	Algeciras (ES), Hamburg (GER), Felixstove (GB), Rotterdam (NL)	4	3	3: 11 (34.0%)	10: 4 (100%)
3^a^	Tanger (MO), Rotterdam (NL), Tilbury (GB), Antwerpen (BE), Le Havre (FR)	5	4	4: 17 (38.0%)	12: 9 (100%)
4	Hamburg (GER), Klaipeda (LT), Riga (LV), Hamburg (GER)	4	6	3: 5 (41.2%)	7: 19 (100%)
5	Hamburg (GER), Bremerhaven (GER), Kaliningrad (RU), Hamburg (GER)	4	5	3: 1 (43.7%)	6: 23 (100%)
6	Hamburg (GER), Kottka (FI), Helsinki (FI), Hamburg (GER)	4	4	2: 18 (32.5%)	8: 11 (100%)
7	Rotterdam (NL), Dublin (IR), Rotterdam (NL)	3	5	4: 19 (57.8%)	8: 7 (100%)
8	Hamburg (GER), Copenhagen (DK), Halmstadt (SE), Szczecin (PL), Hamburg (GER)	5	7	2: 21 (47.9%)	6: 0 (100%)
9	Hamburg (GER), Oslo (NO), Frederikstadt (NO), Larvik (NO), Kristiansand (NO), Hamburg (GER)	6	8	2: 1 (31.2%)	6: 13 (100%)
10	Hamburg (GER), Immingham (GB), Felixstove (GB), Teesport (GB), Grangemouth (GB), Bremerhaven (GER)	6	7	4: 7 (47.2%)	9: 2 (100%)
11	Antwerp (BE), Dublin (IR), Felixstove (GB), Rotterdam (NL)	4	6	3: 13 (54.5%)	6: 12 (100%)
12	Rotterdam (NL), Gothenburg (SE), Rotterdam (NL)	3	4	4: 8 (58.1%)	7: 11 (100%)
13	Rotterdam (NL), Gothenburg (SE), Rotterdam (NL)	3	4	3: 20 (59.7%)	6: 10 (100%)
14	Rotterdam (NL), Arlesund (NO), Orkanger (NO), Salten (NO), Glomfjord (NO), Alesund (NO), Havik (NO), Rotterdam (NL)	8	10	7: 15 (52.2%)	14: 15 (100%)
15	Hamburg (GER), Copenhagen (DK), Halmstadt (SE), Hamburg (GER)	4	6	6: 2 (57.1%)	10: 15 (100%)
16	Hamburg (GER), Klaipeda (LT), Kaliningrad (RU), Gydnia (PO)	4	5	4: 11 (53.0%)	8: 10 (100%)
17^a^	Hamburg (GER), Antwerp (NL)	2	2	1: 0 (40.7%)	2: 11 (100%)
18^a^	Paranagua (BR), Santos (BR)	2	2	0: 23 (37.7%)	2: 13 (100%)
19^a^	Barcelona (ES), Valencia (ES)	2	2	1: 0 (33.3%)	3: 0 (100%)
20^a^	Montevideo (UY), Paranagua (BR)	2	2	1: 0 (40.0%)	2: 12 (100%)
21	Rotterdam (NL), Cork (IR), Rotterdam (NL)	3	4	3: 19 (54.3%)	7: 0 (100%)
22	Hamburg (GER), Bremerhaven (GER), Bremen (GER), Gävle (SE), Oulu (FI), Helsingborg (SE), Hamburg (GER)	7	10	5: 18 (36.5%)	15: 19 (100%)
Total		90	118	78: 3 (43.6%)	179: 6 (100%)

^a^Vessels operating worldwide with episodic extended sea voyages; these vessels were excluded from the total presentation (only feeder vessels)

*BE* Belgium, *BR* Brazil, *DK* Denmark, *ES* Spain, *FI* Finland, *FR* France, *GB* Great Britain, *GER* Germany, *IR* Ireland, *LT* Lithuania, *LV* Latvia, *MO* Morocco, *NL* Netherlands, *NO* Norway, *PL* Poland, *RU* Russia, *SE* Sweden, *UR* Uruguay

While the large container ships nos. 2, 3, 17–20 were operating on worldwide shipping routes, the other examined vessels only sailed in coastal waters (North Sea and Baltic Sea). This led to a higher number of terminals served for seafarers on vessels in the North Range. Due to the increased frequency of port stays in feeder traffic, there were more opportunities for shore leave that could be used for visiting welfare facilities.

### Interviews with seafarers concerning maritime welfare facilities in ports

**Fig. 1:**
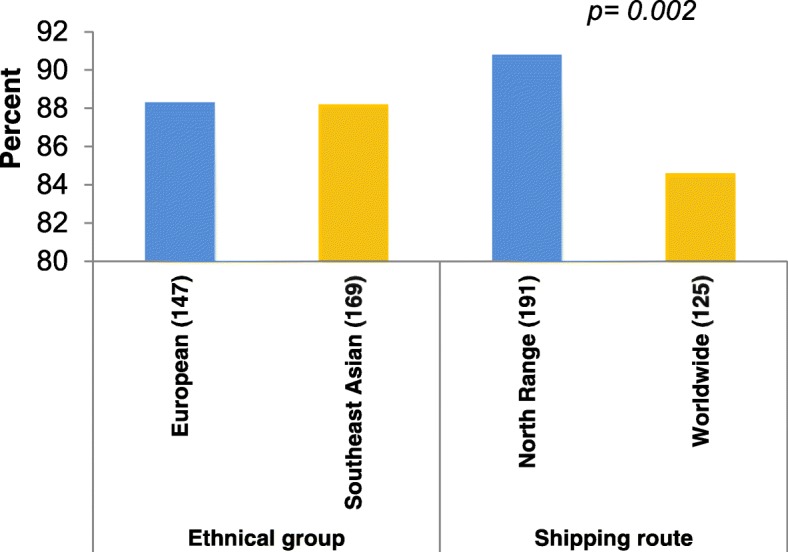
Shore leave opportunities for seafarers

In semi-standardised interviews, 279 seafarers (88.3%) stated that they generally had opportunities to visit welfare facilities during their shore leave. This was significantly more frequent among crews from ships operating in the North Range (which call at more ports than ships in global trade area) (Fig. 1).

**Fig. 2:**
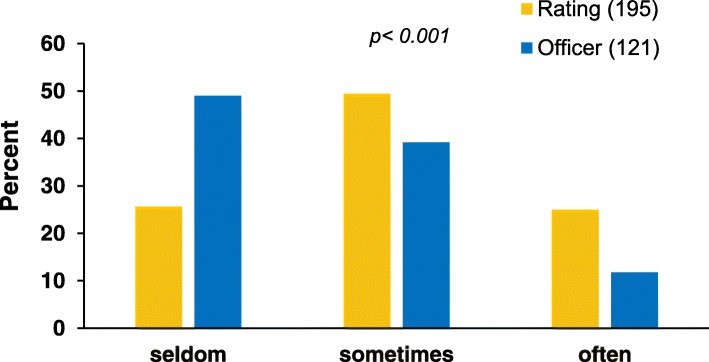
Shore leave frequencies for seafarers depending on ranks

On their last vessels, the interviewed seafarers served, on average, 13 terminals in 30 days (median 11 (range 1–41 terminals)). Within the last voyage, the opportunity for shore leave was only given “often” (that means a minimum of every second port) in 19.0%; 46.2 and 34.4% respectively stated that they “sometimes” (every 3rd - 5th port) or “seldom” (not earlier than every 6th port) had an opportunity for shore leave. Ratings significantly more often indicated having had opportunities for shore leaves (*p* < 0.001) (Fig. 2).

In binary logistic regression (seldom vs. sometimes/often), the officers less often had opportunities for shore leave than ratings (OR 0.40; 95%CI 0.24–0.67). After adjusting for age and duration of stay on board at the time of our interview, the observed OR remained significant (OR 0.35; 95%CI 0.20–0.61).

**Fig. 3:**
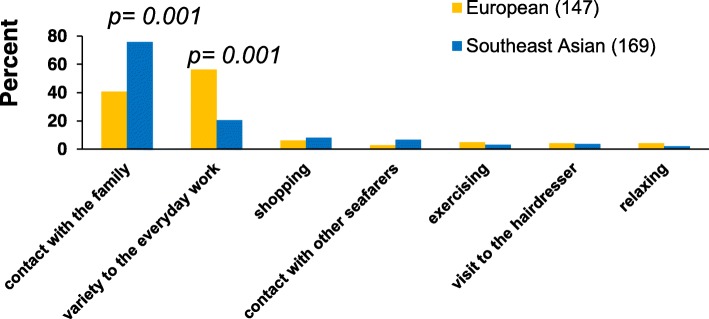
Shore leave reasons for European and Southeast Asian seafarers

In the present study, the main reasons for shore leave were to contact family and friends, to add variety to the everyday work on board, shopping and social contact with other seafarers (Fig. 3). Furthermore, the seafarers were asked to add further important reasons for shore leave. The list was most often completed by visiting religious facilities (especially Southeast Asians: 48.3% vs 3.2%).

A comparison of the ethnic groups shows that contacting the family plays a much more important role for Southeast Asians than for European seafarers (OR 3.79; 95%CI 2.36–6.09), while they less often regarded variety in their everyday life as a very important effect of welfare visits than Europeans (OR 0.23; 95%CI 0.13–0.40). The contact with other seafarers was also especially important for Southeast Asian seafarers. All these associations were independent of the age and the duration of stay on board at the time of our interview.

**Fig. 4:**
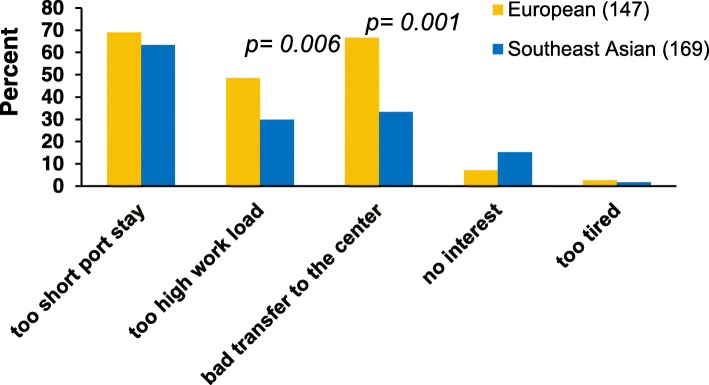
Obstacles to shore leave for European and Southeast Asian seafarers

The main obstacles to shore leave were short port stays, a large amount of work on board and insufficient possibilities for reaching the city centre (Fig. 4). As additional obstacles, 195 deck ratings (61.7%) on feeder ships added that they are often engaged in lashing activities during port stay, and 178 participants (56.3%) stated repetitive shifting of the vessel between different terminals in one port as time-consuming work tasks. Furthermore, 132 seafarers (41.8%) complained about poor information on board about the facilities. In addition, this study reveals that Europeans/officers more often experienced a high workload as an obstacle to shore leave than Southeast Asians/ratings. Moreover, the large amount of work more often constituted an obstacle for younger seafarers (*p* = 0.006) as well as the poorer bus transfer to the welfare facilities for seafarers with a shorter previous stay on board at the time of our interview (*p* < 0.001).

In particular, seafarers on container vessels operating worldwide were hindered in taking shore leave due to a high workload during port stays. Contact with family members and friends was mainly established by seafarers via the Seamen’s Facilities (85.3%), possibilities in the port area (6.7%), public telephones (4.7%) and public facilities in the city centre (e.g. post, internet café, restaurants) (3.3%).

The most important social facilities for seafarers were Seamen’s Missions and religious facilities; half of the participants regarded these facilities as “very important”. This was particularly true among Southeast Asians (representing the majority of the ratings in this study (Table 2). Social institutions with sport facilities also played a bigger role for Southeast Asian seafarers. Crew members on ships operating worldwide attached great importance to visits to bars. Only 16 persons mentioned shopping opportunities for satisfying individual needs as being very important (Table 2).

**Table 2:** Rating “very great importance” of different maritime welfare facilities

Maritime welfare facilities		Ethnic Group		Ranking		Shipping route	
	*Overall study sample n (% of all evaluations)*	*Euro-pean (147)*	*South-east Asians (169)*	*Rating (195)*	*Officer (121)*	*North Range (191)*	*World-wide (125)*
Seamen’s Missions	87 (33.5%)	22 (17.3%)	65 (48.9%) ***	64 (40.3%)	23 (22.8%) ***	53 (29.8%)	34 (41.5%)
Religious facility	80 (31.1%)	9 (7.2%)	71 (53.8%) ***	68 (43.0%)	12 (12.1%) ***	59 (33.1%)	21 (26.6%)
Sport Club	33 (13.2%)	9 (7.3%)	24 (19.0%) **	23 (15.2%)	10 (10.1%)	29 (16.4%)	4 (5.5%) ***
Bar	29 (11.3%)	12 (9.4%)	17 (13.2%)	23 (14.8%)	6 (5.9%) *	6 (3.4%)	23 (29.1%) ***
Shops	16 (4.8%)	10 (6.5%)	6 (3.4%)	9 (4.4%)	7 (5.7%)	7 (3.5%)	9 (6.8%)

Chi^2^-test: *p ≤ 0.05 and > 0.01; **p ≤ 0.01 and > 0.001; ***p ≤ 0.001

## Discussion

This study evaluated for the first time the proportions of time during the voyage of 18 feeder vessels and demonstrated that the port stay lasted for 43.6% of the entire voyage duration. This time slice can be regarded as sufficient for seafarers to leave the vessel in port in order to visit welfare facilities; this applies especially to feeder ships. In contrast, longer time periods have to be considered on board vessels operating worldwide due to the sea passage across the oceans and hence fewer port calls. Thus, the seafarers on these vessels have significantly less opportunities to visit welfare organisations.

According to the study findings, major reasons for shore leave were to contact family and friends, shopping and social contacts with other seafarers. During their stay on board, crew members are involved in an almost closed social system with a hierarchical role structure [14]. Consequently, face-to-face communication is limited to the crew members for a long time. This means very one-sided and reduced communication on board. Therefore, contact opportunities and new communication partners outside of the vessel are very important to preserve the crews’ social competence [15]. Interestingly, this study proves that the duration of previous stay on board at the time of our interview does not influence the frequency of visits to welfare facilities. Thus, an exploration of the seafarers’ behaviours to visit welfare facilities was independent of the duration of stay on board.

Due to short stays in port, terminals far away from the city centres and security measures in ports, maritime welfare facilities, especially Seamen’s Missions next to the terminal, are assessed as an important meeting point for seafarers [13]. In this study many seafarers additionally appreciated the fact that the facilities represent a change from their everyday work on board. Importantly, this enables the crew members to leave their roles in the hierarchical ship’s operation at least for a short time and to use different contact, communication and relaxing opportunities that go beyond digital communication with the family. It has repeatedly been described that this relieving change in role can lead to relaxation, attention and devotions which are not present in normal on-board operations [9, 16, 17]. According to Palmer and Murray [10], especially after extreme stress situations, unfair treatment or in a personal crisis, welfare facilities/seamen’s facilities are important places where the affected seamen can find understanding, support and, in many cases, help.

The present study demonstrated that sport facilities are of less significance for crew members on ships operating worldwide. This is likely due to the fact that these types of ships are often already equipped with a sport facility. The greater importance seafarers in global trade place on visiting bars may express a higher need for social contacts beyond the ship after long voyages.

Moreover, this study reveals that 50% of the Southeast Asian seafarers and approx. 12% of the Europeans regarded Seamen’s Missions and religious facilities as very important. This culture-specific difference typically reflects a depth of spirituality among Asian crew members, on the one hand, and the desire for closer contacts outside of the social constraints on board, on the other hand.

The main obstacles to shore leave were short port stays, a large amount of work on board, insufficient possibilities for reaching the city centre and poor information about the welfare facilities. While the shortness of port stay − as an unchangeable deliverable − was attributable to economic reasons, the other aspects address various competent bodies:

Firstly, the seafarers’ high workload during port stay should be reduced. According to this study, the high workload results from repetitive shifting of the vessel between different terminals in one port. This statement was also confirmed by other references [18, 19]. The present study objectified the serving of 41 terminals in 30 days in an extreme case. Furthermore, many participants mentioned container lashing as a high workload for deck ratings. It has been described that this task is very time-consuming and often hampers crew members from visiting welfare facilities [20]. The lashing of cargo should be conducted by external shoreside personnel [21] in order to relieve seafarers during port stays and to offer them the opportunity to visit welfare facilities. Correspondingly, according to the contracts with the International Transport Workers’ Federation (ITF 2018), handling and lashing tasks should be performed by land-based workers (so-called lashing workers). Besides the time consumed by lashing activities and due to this psychophysically stressful work, the seafarers are often exhausted and have no energy to enjoy leisure time activities ashore during port stays [22].

Additionally, officers stated that they less often had sufficient opportunities for shore leave, irrespective of their age and their previous stay on board (OR 0.35; 95%CI 0.20–0.61). In port, officers have a high workload as they are often intensively involved in the planning and control of loading and unloading operations with a high degree of responsibility, which could also lead to increased fatigue [23–25].

Secondly, compared to Southeast Asians the Europeans more often mentioned insufficient possibilities for transfer to the city centre as barriers to shore leave. This may reflect their perceptions of high job-related stress levels or a state of great fatigue among this rank group. As operational action, the port or terminal operator should try to reduce the number of terminals to be called at. This would lead to a lower frequency of mooring manoeuvres and consequently enable all seafarers on board to make greater use of leisure facilities. Furthermore, more attention should be paid to the accessibility of the welfare facilities by bus transfer.

Thirdly, several seafarers complained about the poor information regarding the facilities. Thus, a targeted distribution of information via Internet about their regular offerings, the city, the port and the current local events is recommended. On the other hand, it would be helpful for the crews if the ship’s management informed the mission in good time of the ship’s visit and if a crew member had contact needs or personal problems.

### Strengths and limitations

As a limitation, the present study could not cover the different port structures with regard to the possible availability and accessibility of welfare facilities. It has not focused on the potential impact of ship and port security measures according to the International Ship and Port Facility Security Code (ISPS) for shore leave to visit the welfare facilities. This is the first study on maritime recreational facilities, and it was also not concerned with the size, equipment and temporal availability of the world’s welfare institutions.

As a strength of this study, the high participation rate of over 86.6% in the study proves the great importance of welfare facilities for seafarers. A key feature of the study and its results was the performance of a standardised individual interview with the crew member on board as a qualitative procedure. Interviewing is the most common format of data collection in qualitative research [26]. According to Wynn and Money [27], particularly qualitative research and procedures provide a valuable insight into human actions, beliefs and values in occupational medicine. Accordingly, the qualitative method of the study was able to identify wishes and significance as well as occupational and ethnic differences in the use of welfare facilities by seafarers. In a controllable individual interview, different language comprehension and cultural differences within a multicultural crew could be taken into account [28]. The interview with the crew member in his familiar working and living situation during the ship’s operation and his anonymity within the scope of the investigation were decisive conditions for reliable statements.

## Conclusions

Maritime welfare facilities constitute an important refuge for seafarers beyond their ships to recover from demanding work and to “recharge their batteries”. Measures should be taken to facilitate visits to maritime welfare facilities.

## Abbreviations

CI: confidence interval
ISPS: International Ship and Port Facility Security Code
ITF: International Transport Workers’ Federation
OR: odds ratio
